# Cervical cancer survival prediction by machine learning algorithms: a systematic review

**DOI:** 10.1186/s12885-023-10808-3

**Published:** 2023-04-13

**Authors:** Milad Rahimi, Atieh Akbari, Farkhondeh Asadi, Hassan Emami

**Affiliations:** 1 Department of Health Information Technology and Management, Medical Informatics, School of Allied Medical Sciences, Shahid Beheshti University of Medical Sciences, Tehran, Iran; 2 Obstetrics and Gynecology, Cancer Research Center, Shahid Beheshti University of Medical Sciences, Tehran, Iran; 3 Department of Health Information Technology and Management, Health Information Management, School of Allied Medical Sciences, Shahid Beheshti University of Medical Sciences, Tehran, Iran; 4 Department of Health Information Technology and Management, Information Technology, School of Allied Medical Sciences, Shahid Beheshti University of Medical Sciences, Tehran, Iran

**Keywords:** Cervical Cancer, Machine Learning, Deep Learning, Survival Prediction

## Abstract

**Background:**

Cervical cancer is a common malignant tumor of the female reproductive system and is considered a leading cause of mortality in women worldwide. The analysis of time to event, which is crucial for any clinical research, can be well done with the method of survival prediction. This study aims to systematically investigate the use of machine learning to predict survival in patients with cervical cancer.

**Method:**

An electronic search of the PubMed, Scopus, and Web of Science databases was performed on October 1, 2022. All articles extracted from the databases were collected in an Excel file and duplicate articles were removed. The articles were screened twice based on the title and the abstract and checked again with the inclusion and exclusion criteria. The main inclusion criterion was machine learning algorithms for predicting cervical cancer survival. The information extracted from the articles included authors, publication year, dataset details, survival type, evaluation criteria, machine learning models, and the algorithm execution method.

**Results:**

A total of 13 articles were included in this study, most of which were published from 2018 onwards. The most common machine learning models were random forest (6 articles, 46%), logistic regression (4 articles, 30%), support vector machines (3 articles, 23%), ensemble and hybrid learning (3 articles, 23%), and Deep Learning (3 articles, 23%). The number of sample datasets in the study varied between 85 and 14946 patients, and the models were internally validated except for two articles. The area under the curve (AUC) range for overall survival (0.40 to 0.99), disease-free survival (0.56 to 0.88), and progression-free survival (0.67 to 0.81), respectively from (lowest to highest) received. Finally, 15 variables with an effective role in predicting cervical cancer survival were identified.

**Conclusion:**

Combining heterogeneous multidimensional data with machine learning techniques can play a very influential role in predicting cervical cancer survival. Despite the benefits of machine learning, the problem of interpretability, explainability, and imbalanced datasets is still one of the biggest challenges. Providing machine learning algorithms for survival prediction as a standard requires further studies.

## Introduction

Cervical cancer is the fourth most common cancer in the female reproductive system and the seventh most common cancer worldwide. There is a higher likelihood of cancer tumors growing in areas where endocervix cells become exocervix cells or near the Squamocolumnar Junction (SCJ). Cervical cancer is one of the main factors related to the death of females worldwide [1]. According to the World Health Organization (WHO) cervical cancer report in 2020, there were about 604,127 diagnosed cases and 341,831 deaths worldwide, of which 1,056 diagnosed cases and 644 deaths occurred in Iran [2]. Sexually transmitted diseases, multiple partners, smoking, weak nutrition, and the immune system play a role in the growth and development of cervical cancer [3]. An important risk factor for cervical cancer is the persistence of human papillomavirus (HPV), especially genotypes 16 and 18 [4]. Although about 90% of human papillomavirus infections heal by themselves within two years, some may also lead to the growth of cancerous masses in the cervix [5, 6]. Diagnosing a cancerous mass in the early stages increases the patient’s chance of survival and treatment. In late diagnosis, the possibility of complete recovery of the patient decreases [7]. Cervical cancer is entirely preventable and treatable if pre-cancer symptoms are identified at an early stage. The pap smear is frequently used for cervix medical diagnosis to track cervical cancer. A few cervical cell samples are taken, a cell smear is made, the cells are examined under a microscope for abnormalities, and the result is a diagnosis of the cervical condition [8]. Physicians consider the patient's chance of survival to guide their treatment plan.

Survival prediction is a set of statistical methods for data analysis, where the outcome variable is the time to an event. In other words, survival prediction is calculated by considering the time between exposure to the event and the occurrence of the event [9]. According to the American Society of Clinical Oncology (ASCO), the average 5-year overall survival rate for cervical cancer is 66%, i.e., about 66% of people diagnosed with cervical cancer today will survive for at least the next five years. The best treatment method for each patient can be adopted by evaluating the patient’s clinical and treatment data to accurately predict the patient’s survival. Researchers have often used classical statistical methods such as non-parametric, parametric, and semi-parametric (COX) tests to predict survival [10]. In recent years, artificial intelligence algorithms, with their impressive capabilities, have been in fierce competition with statistical tests and have grown significantly in survival prediction.

Big data are being generated and stored with the rapid growth of digital technologies in healthcare and the evolution of electronic health records (EHR) [11]. Classical statistical methods often focus on the relationship between dependent variables to achieve the final result, but machine learning algorithms can learn hidden patterns in data. Machine learning algorithms do not require implicit assumptions and can manage non-linear relationships between variables [12]. Machine learning makes computers intelligent without directly teaching them how to make decisions and solve problems [13]. Today, machine learning algorithms have been studied and developed in the diagnosis, prognosis, and prediction of the occurrence of many diseases [14], which performed very well in dealing with Big data [15].

This study aimed to evaluate published studies on machine learning algorithms in predicting the survival of patients with cervical cancer, considering overall, disease-free, and progression-free survival.

## Materials and methods

This systematic review examined original articles that used machine learning algorithms to predict the survival of patients with cervical cancer and discovered knowledge.

### Study selection

**Fig. 1:**
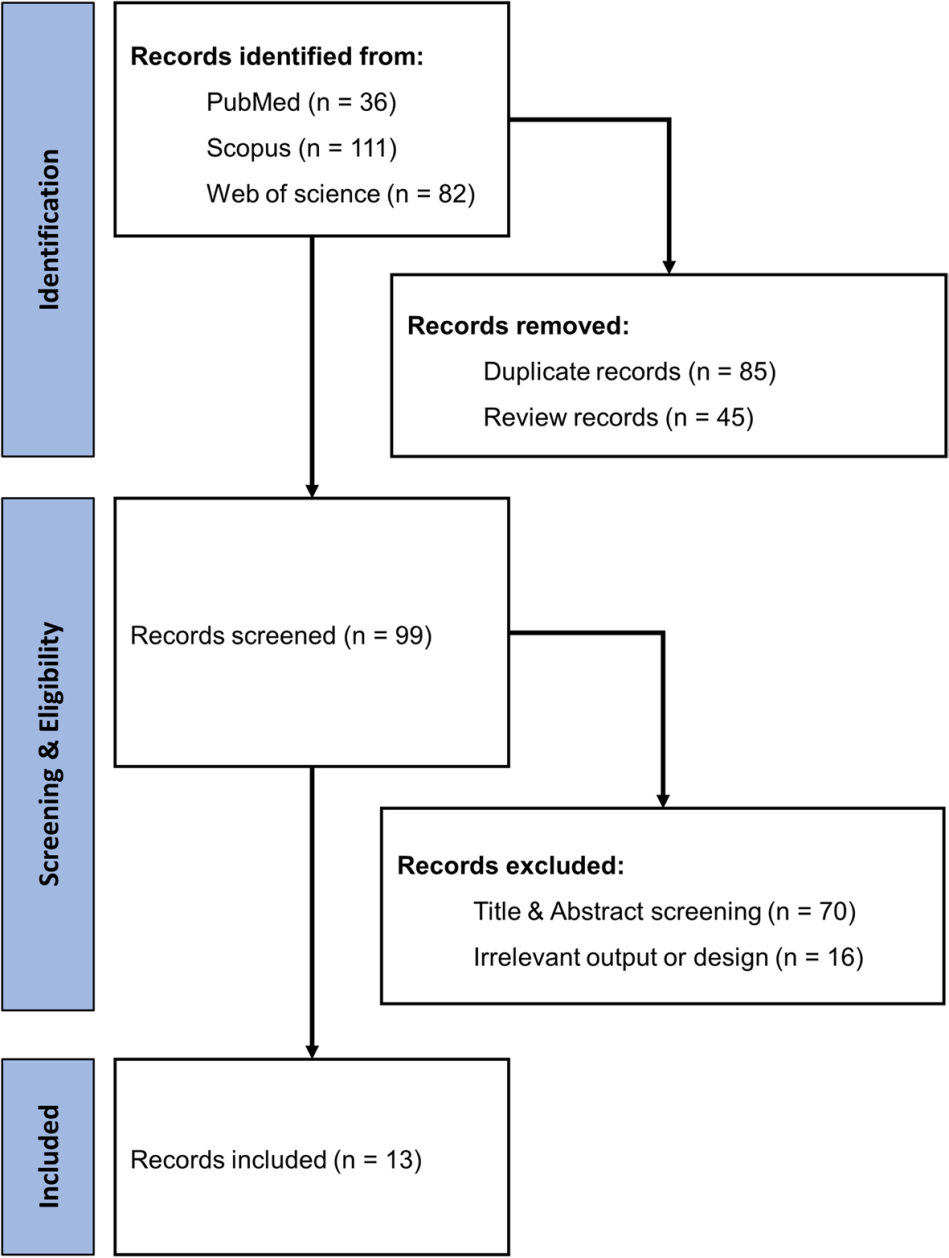
Description: Flow diagram of the study identification and selection process, following Preferred Reporting Items for Systematic Reviews and Meta-Analyses (PRISMA) guidelines

The article selection method was based on the Preferred Protocol for Systematic Reviews and Meta-Analysis (PRISMA) and the retrieved articles were imported into Excel software. The first search returned 229 articles, then 45 review articles and 85 duplicate articles were removed. A total of 99 items remained for screening based on the eligibility criteria. During the screening process, 70 articles were excluded by title and abstract verification, and 16 articles were excluded based on method, results, or study design nature. The screening process was performed twice to reduce errors. Any discrepancies were resolved through discussions with the second and third authors. Finally, 13 articles were thoroughly examined and included in the study (Fig. 1).

### Search strategy

Articles published until October 1, 2022, were collected from three electronic databases, PubMed, Scopus, and Web of Science, and the search query consisted of three basic parts. The first part was about cervical cancer, which included two keywords of "cervical cancer" and "Uterine Cervical Neoplasms". The second part was about predicting survival with one keyword named "Survival", and the third part was about artificial intelligence with three keywords, including "Machine learning", "Deep learning", and "Artificial Intelligence." Details are available in Table 1.

**Table 1:** Keywords and search strategy in three databases: PubMed, Scopus, and Web of Science

#1: (Uterine Cervical Neoplasms OR Cervical Cancer) #2: (Survival) #3: (Machine Learning OR Deep Learning OR Artificial Intelligence)
Search strategy: #1 AND #2 AND #3
PUBMED: (Uterine Cervical Neoplasms[Title/Abstract] OR Cervical Cancer[Title/Abstract]) AND (Survival[Title/Abstract]) AND (Machine Learning[Title/Abstract] OR Deep Learning[Title/Abstract] OR Artificial Intelligence[Title/Abstract])
SCOPUS: (TITLE-ABS-KEY(Uterine Cervical Neoplasms) OR TITLE-ABS-KEY(Cervical Cancer)) AND (TITLE-ABS-KEY(survival)) AND (TITLE-ABS-KEY(Machine Learning) OR TITLE-ABS-KEY(Deep Learning) OR TITLE-ABS-KEY(Artificial Intelligence))
WEB OF SCIENCE: (TS = (Uterine Cervical Neoplasms) OR TS = (Cervical Cancer)) AND (TS = (Survival)) AND (TS = (Machine Learning) OR TS = (Deep Learning) OR TS = (Artificial Intelligence))

### Inclusion and exclusion criteria

This study included original articles and full English text, which used machine learning algorithms as predictive models for cervical cancer survival.

Books, review articles, meta-analyses, case reports, posters and case studies were filtered. In addition, articles that did not sufficiently focus on the implementation of machine learning algorithms, cervical cancer, and model outputs were excluded in the screening section. All entry and exit criteria are listed in Table 2.

**Table 2:** Inclusion and exclusion criteria for articles in the study

Inclusion criteria	Exclusion criteria
Full text access	Book
Full text English	Review and Meta-analysis
Original articles	Letters to the editor
	Short article and Poster
	Case report

## Results

From the initial search results, 229 articles were found, of which only 13 articles met the study criteria and were included in the study for further investigation. All included articles were retrospective and used machine learning algorithms as modeling to predict cervical cancer survival.

### Characteristics of studies

Most of the imported articles were published from 2018 onwards, and the last was from 2022 (Table 3). Table 4 provides additional information and a general view of the included studies. A total of eight articles were performed in Asia [16–23], four in Europe [24–27], and one in the United States [28]. Generally, eight articles on overall survival (OS) [17, 19–21, 23, 26–28], six articles on disease-free survival (DFS) [16, 18, 21–24], and three articles on survival progression-free (PFS) [19, 25, 28] were used to predict the survival of patients with cervical cancer. Moreover, two articles were excluded from the study due to the use of machine learning algorithms only as a tool for feature selection [29, 30].

**Table 3:** Extracted characteristics of the included articles

Author, Year	Country	Data source	# Samples	Hyperparameter tuning	Pre-processing	Feature selection	Survival	Data types	ML algorithms	Validation	Evaluation
Liang, 2022 [17]	China	SEER (2010 to 2015)	14946	No	Yes	Yes	OS	Clinical	LR	Internal	C-index
Ding, 2021 [20]	China	The cancer Genome Atlas	542	No	Yes	Yes	OS	Molecular Clinical	SVM	Internal	AUC
Obrzut, 2017 [27]	Poland	Rzeszow State Hospital (1998 to 2001)	117	No	Yes	No	OS	Clinical	PNN MLP GEP SVM RBFNN K-Means	Internal	Accuracy Sensitivity Specificity AUC
Carlini, 2022 [26]	Italy	IRCCS University Hospital	85	Yes	Yes	Yes	OS	PET/CT	RF	Internal	C-index
Ferreira, 2021 [24]	Belgium	Liege University Hospital (2010 to 2016)	158	Yes	Yes	Yes	DFS	PET/CT Clinical	RF SVM NB LR	External	AUC F1-score Precision Sensitivity
Takada, 2020 [16]	Japan	Chiba Hospital (2012 to 2016)	107	No	Yes	Yes	DFS	Clinical MRI	RF	Internal	AUC
Senthilkumar, 2021 [18]	India	GEO	300	Yes	Yes	Yes	DFS	Molecular	EL CoxLasso	External	Precision F1-score Accuracy Sensitivity
Shen, 2019 [22]	Taiwan	(2009 to 2015)	142	No	Yes	Yes	DFS	Clinical PET/CT	DL	Internal	Sensitivity Specificity Accuracy PPV NPV
Arezzo, 2021 [25]	Italy	University of Bari, (2010 to 2018)	92	Yes	No	Yes	PFS	Clinical MRI	LR RF KNN	Internal	Accuracy TPR Precision AUC
Guo, 2021 [21]	China	multi-center (2006 to 2017)	5112	Yes	No	Yes	OS; DFS	Clinical	GBDT RF	Internal	C-index MAE
Chen, 2022 [23]	China	Nanfang Hospital (2009–2016)	251	No	Yes	Yes	OS; DFS	Clinical WSI	DL	Internal	C-index AUC
Matsuo, 2019 [28]	USA	California Medical Center (2000 to 2014)	768	Yes	No	Yes	OS; PFS	Clinical	CoxBoost CoxLasso RF DL	Internal	C-index MAE
Kim, 2021 [19]	Korea	Multi-center (2000 to 2018)	1056	No	Yes	Yes	OS; PFS	Clinical	LR HL	Internal	AUC

*PNN* Probabilistic neural network, *ANN* Artificial neural network, *MLP* Multilayer perceptron network, *GEP* Gene expression programming classifier, *SVM* Support vector machines, *RBFNN* Radial basis function neural network, *RF* Random Forest, *LR* Logistic regression, *NB* Naïve bayes, *DL* Deep learning, *KNN* K-nearest neighbors, *DVH* Dose-volume Histogram, *OS*, Overall survival, *DFS* Disease-free survival, *PFS* progression-free survival, *WSI* Whole slide image, *EL* Ensemble learning, *HL* Hybrid learning

**Table 4:** Classification of the features of the included articles

Characteristics	Categories	Number (n)		
		OS	DFS	PFS
Location	Asia	5 [17, 19–21, 23]	5 [16, 18, 21–23]	1 [19]
	Europe	2 [26, 27]	1 [24]	1 [25]
	USA	1 [28]	-	1 [28]
Dataset sources	Hospitals	6 [19, 21, 23, 26–28]	5 [16, 21–24]	3 [19, 25, 28]
	SEER	1 [17]	-	-
	TCGA	1 [20]	-	-
	GEO	-	1 [18]	-
Dataset privacy	Public	2 [17, 20]	1 [18]	-
	Private	6 [19, 21, 23, 26–28]	5 [16, 21–24]	3 [19, 25, 28]
Data source	Single	6 [17, 20, 23, 26–28]	5 [16, 18, 22–24]	2 [25, 28]
	Multiple	2 [19, 21]	1 [21]	1 [19]
Preprocessing	Yes	6 [17, 19, 20, 23, 26, 27]	5 [16, 18, 22–24]	1 [19]
	No	2 [21, 28]	1 [21]	2 [25, 28]
Feature selection	Yes	7 [17, 19–21, 23, 26, 28]	6 [16, 18, 21–24]	3 [19, 25, 28]
	No	1 [27]	-	-
# Models	One	4 [17, 20, 23, 26]	3 [16, 22, 23]	-
	Two or more	4 [19, 21, 27, 28]	3 [18, 21, 24]	3 [19, 25, 28]
Models type	RF	3 [21, 26, 28]	3 [16, 21, 24]	2 [25, 28]
	LR	1 [19]	2 [17, 24]	2 [19, 25]
	SVM	2 [20, 27]	1 [24]	-
	DL	2 [23, 28]	2 [22, 23]	-
	H&E L	2 [19, 21]	2 [18, 21]	1 [19]
Validation	Internal	8 [17, 19–21, 23, 26–28]	4 [16, 21–23]	3 [19, 25, 28]
	External	-	2 [18, 24]	-
Evaluation metrics	AUC	4 [19, 20, 23, 27]	4 [16, 19, 23, 24]	1 [25]
	C-index	5 [17, 19, 21, 23, 26]	2 [21, 23]	1 [19]
	Sensitivity	1 [27]	3 [18, 22, 24]	-
	Precision	-	2 [18, 24]	1 [25]
	Specificity	1 [27]	1 [22]	-
	Accuracy	1 [27]	2 [18, 22]	1 [25]
	F1-score	-	2 [18, 24]	-
	MAE	2 [21, 28]	1 [21]	1 [28]
	NPV / PPV	-	1 [22]	-
Data types	Clinical	5 [17, 19, 21, 27, 28]	1 [21]	2 [19, 28]
	Image	1 [26]	-	-
	Molecular	-	1 [18]	-
	Clinical + Image	1 [23]	4 [16, 22–24]	1 [25]
	Clinical + Molecular	1 [20]	-	-

*TCGA* The cancer Genome Atlas, *GEO* Gene Expression Omnibus, *SEER* Surveillance, Epidemiology, and End Results, *RF* Random Forest, *SVM* Support Vector Machine, *LR* Logistic regression, *DL* Deep Learning, *H&E* L Hybrid and Ensemble learning, *MAE* Mean absolute error

#### Database information

Ten articles used hospital and clinic datasets [16, 19, 21–28], and three articles each used the cancer genome atlas [20], SEER [17], and Geo [18]. The datasets used in the three articles were more detailed and open to public access [17, 18, 20], but private datasets were used in the other ten articles. The maximum and minimum sizes of the datasets used for modeling were 14,946 and 85 records, respectively, and the datasets had more than 1000 records only in three articles [17, 19, 21].

#### Data preprocessing

A total of 11 articles used data preprocessing techniques [16–26], and three mentioned missing data [18, 19, 25]. Selected approaches to handle missing data included record deletion, multiple imputations, and the nearest neighbor algorithm. The feature selection approach was used in all the articles except article [27], but only eight articles specified the details [16, 18, 20, 21, 23–26]. Logistic regression [24], Naive Bayes [24], Random Forest [24], Genetic algorithm [26], lasso [17, 18, 25, 27], k-means [19, 20], Support vector machine [18, 19, 26, 28], AdaBoost [18], Elastic-net [23], recurrent feature elimination (RFE) [16, 25], and deep learning [22, 23, 28] were among the algorithms used for feature selection and extraction. Two articles mentioned the management of outlier data [16, 20], but only one provided more details [16].

Imbalanced data in the dataset causes a lack of generalizability in the model and is considered a serious challenge [31]. The challenge of unbalanced data in the dataset was discussed in two articles [25, 26], and the RF cost-sensitive method was used to overcome this challenge in one article [25].

#### Data modeling

The model was calibrated in three articles [16, 18, 25], but the work details were not provided. Hyperparameter tuning was used in model training in six articles, but only four shared the work details [18, 24, 25, 28].

Six articles used only one machine learning algorithm to build the model [16, 17, 20, 22, 23, 26]. Further, two or more machine learning algorithms were used in seven articles, and their output was compared with each other [18, 19, 21, 24, 25, 27, 28]. The most frequent machine learning algorithms were random forest, logistic regression, support vector machine, deep learning, and ensemble and hybrid learning.

#### Model validation

The selected articles were based on internal validation in 11 articles and external validation in two articles [18, 24]. Most of the studies related to internal validation used the cross-validation method.

The most common criteria for evaluating the algorithm performance in the articles were the model AUC from 0.40 to 0.99 in seven articles, regardless of the type of survival. C-index was 0.39 to 0.94 in 5 articles, and the accuracy was 0.61 to 0.92 in 4 articles. In three articles, sensitivity and F1-score were 0.20 to 0.97 and 0.22 to 0.92, respectively. More details were shown in Table 5.

**Table 5:** Classification of the used evaluation criteria into types of survival from the lowest to the highest

Evaluation method	OS		DFS		PFS	
	Min	Max	Min	Max	Min	Max
AUC	0.40	0.99	0.56	0.88	0.67	0.81
C-index	0.39	0.94	0.41	0.89	0.69	0.79
Sensitivity	0.75	0.97	0.20	0.93	-	-
Specificity	0.0	0.60	0.93	0.93	-	-
Precision	-	-	0.33	91.14	76.5	80.1
Accuracy	0.61	0.89	0.84	0.92	0.73	0.84
F1-score	-	-	0.22	0.92	-	-
Mean Absolute Error	21.18	39.2	11.24	12.43	28.8	29.3

Table 5 description: All of the articles that employed the selected criteria were split according to the kind of survival, and the minimum and maximum rates for each criterion were then shown

Regarding articles with more than one model, ensemble and hybrid models in 3 articles [18, 19, 21], random forest in 3 articles [24–26], logistic regression [17], and deep learning [28] in 1 article had the best performance.

### Important variables

Clinical tabular data were used as model inputs in 11 articles [16, 17, 19–25, 27, 28], which were the only model inputs in five articles [17, 19, 21, 27, 28]. Image-based data was used [16, 22–26] in six articles, one of which applied the machine learning model trained only with images [26]. In two articles, molecular data were used to predict survival [18, 20]. According to the output of all survival prediction models, cancer stage variables, histology, treatment method, and tumor-related information have significantly affected cervical cancer survival prediction. The important variables extracted from the included articles are shown in Table 6.

**Table 6:** Influential variables in predicting types of survival extracted from articles

Selected Features	OS (n)	DFS (n)	PFS (n)
FIGO Stage	5 [17, 20, 21, 23, 25]	4 [16, 21–23]	2 [19, 25]
Heart Rate	1 [25]	-	1 [25]
Laboratory test	1 [25]	-	1 [25]
Treatment type	3 [17, 21, 25]	1 [21]	2 [19, 25]
Race/ethnicity	2 [20, 25]	-	1 [25]
Hypertension	-	-	1 [25]
Histopathology	4 [19–21, 23]	5 [16, 21–24]	1 [25]
Age	3 [17, 20, 23]	3 [22–24]	1 [25]
Height	1 [20]	-	-
Tumor Size	3 [19, 21, 23]	3 [16, 21, 23]	-
Lymph Node metastasis	3 [19, 21, 23]	3 [22–24]	1 [19]
positive lymph node numbers	1 [23]	1 [23]	-
Lymph vascular space invasion	1 [23]	1 [23]	-
BMI	-	-	1 [25]
HPV	1 [20]	-	-

*BMI* Body Mass Index, *HPV* Human Papillomavirus

Table 6 description: From the entered articles, all variables that could reliably predict cervical cancer survival were retrieved, categorised, and then displayed according to the type of survival.

## Discussion

A systematic review of 229 articles resulted in the inclusion of 13 articles. The selected articles contained qualitative and quantitative information about predicting and analyzing the survival of cervical cancer patients using machine learning algorithms. The number of articles using machine learning algorithms to predict cervical cancer survival was few. Studies related to all three types (overall survival, disease-free survival, and progression-free survival) were inevitably included in the study due to the variation in survival and the small number of studies specific to each type of survival.

The three included studies that used open-access databases were more transparent and competitive in preprocessing and model building. Multiple researchers can analyze open-access databases to discover the most valuable features and the best machine-learning model for that particular dataset. Another essential thing even mentioned in the article [32] was the correlation of the model output with the data of a specific geographical environment and the change of medical prescriptions over time. Generalizability and the time interval between data collection and modeling can be evaluated in the applicability of the model output. Databases with open access were more suitable and valuable for studying and predicting survival.

The included articles used datasets with different sizes and types for modeling. The largest dataset included in the study was related to the article [17], with 14,946 clinical tabular data and C-index (0.86). The smallest dataset included in the study is related to the article [26] with 85 image data records (PET/CT) and C-index (0.77). Image datasets had fewer records than other datasets among the imported articles. According to the reports of (Illia Horenko) [33], small datasets used in model training often cause overfitting of the model and reduce the model’s capacity for generalization. Image datasets sometimes make the model more accurate than tabular data, which can be caused by the power of image processing algorithms [34]. Feature extraction, feature selection, transfer learning, fine-tuning, augmentation, object segmentation, and object detection were the most critical advantages of image processing algorithms [34–36]. In addition to the cases mentioned, convolutional neural networks obtained valuable results on 3D images [37]. Recently, medical image datasets have been used to predict the survival of patients. However, larger image datasets and more optimal convolutional neural network structures should reach a robust model.

Only two of the articles included in this study had external validation. Article [18] with molecular data and the other article [24] with the combination of clinical tabular data and images (PET/CT) obtained precision of 0.82 and 0.42 respectively. The model’s generalizability is more reliable in external validation due to the use of different data. Most included articles used the five-fold cross-validation method for internal validation. Cross-validation is a resampling method for evaluating a model with limited data [38]. The advent of open-access datasets and standard databases of medical data has made it more feasible to evaluate models using external validation methods.

Data wrangling and preprocessing play an essential role in modeling and model output. Medical datasets often include noise, redundant data, outliers, missing data, and irrelevant variables [39]. Hoeren mentioned that the actual value of data lies in its usability [40], and data quality is the most critical concern in model training. Data cleaning is one of the essential solutions in the data preprocessing stage for reducing errors, preventing model bias caused by dirty data, and obtaining the best results [41]. Therefore, data preprocessing such as cleaning, transformation, reduction, and integration, should be conducted properly, which includes 70–80% of the training and model workload [42]. All the included studies paid attention to this principle.

Among all the included articles, six used hyperparameter tuning and feature selection methods in their study [18, 21, 24–26, 28]. Studies often used hyperparameter tuning and feature selection to avoid overfitting or to achieve high-accuracy models [24, 25]. According to articles [25, 32], selecting appropriate modeling variables directly affected the model’s output. Therefore, feature selection, extraction, reduction, and engineering are necessary to reach an ideal model. Hyperparameter tuning is one of the essential steps in the model-building pipeline, which can produce a model with high accuracy by finding the most optimal input parameters. Most of the entered studies used the Grid search method for this operation. Considering that feature selection in convolutional neural networks is done automatically, having background knowledge can enhance the model’s reliability. Approaches such as Bayesian Optimization and Evolutionary algorithms like Genetic Algorithms [26] and Artificial Fish Swarm [18] can be more suitable approaches for hyperparameter tuning and feature selection.

Recently, the use of Hybrid and Ensemble models has increased in the medical field, especially in predicting survival. Three of the included studies that used the abovementioned methods to predict survival have obtained acceptable accuracy and precision [18, 19, 21]. Random forest (RF) and Extreme Gradient Boosting (XGBoost) models are also among Ensemble learning (EL) algorithms [26]. Developing and optimizing machine learning models using hybrid and ensemble techniques continuously improve computational aspects, performance, generalizability, and accuracy [43]. Ensemble models, like deep learning algorithms, have spontaneous feature selection ability. In these two Ensemble and Hybrid learning methods, several models with weak learners are trained to solve a specific problem and combined to achieve better results [44].

Most studies have used a combination of clinical, imaging, and molecular data to predict survival to achieve greater accuracy in training machine learning models. Articles [22–25] used a combination of clinical data types with more accuracy and reliability. Most articles that used composite data to predict cervical cancer survival occurred from 2021 onwards. Random forest and deep learning were the most used in mixed data modeling. All types of patient data, with the help of artificial intelligence, can play a significant role in Precision Medicine.

With recent advances in artificial intelligence, deep learning algorithms have undeniably gained power as well. Deep learning algorithms are able to recognize patterns from large, extensive and heterogenous data. They have also provided an admirable ability to process image, video, text, audio and signals [45]. According to comparative studies, it has been determined that artificial intelligence has a better performance than classical statistics [45]. With the daily advancement of technologies and the rapid expansion of artificial intelligence science, we will see the use of transformers [46], meta learning [47] and quantum machine learning [48] in medical data processing in the near future. Nevertheless, solutions to the questions of interpretability and explainability should be considered together with the immense potential of AI in health research [49].

## Conclusions

Recording and storing patient information has become easy and is overgrowing due to the growth and improvement of hospital information systems (HIS) and electronic health record systems (EHRs). Classical statistical models such as Cox are used in many survival studies but are no longer compatible with many medical data. Today, machine learning algorithms have become a focal point in research and development because of their unique abilities in pattern recognition in data, feature selection and extraction, and great power in medical image processing.

Most of the survival articles of the last few years have used machine learning algorithms to predict the survival of cervical cancer patients. Combining heterogeneous multidimensional data with machine learning techniques could affect the prediction of cervical cancer survival. The low or lack of explainability in machine learning algorithms has prevented the official use of artificial intelligence models in health. Machine learning is more accurate than other statistical methods in predicting the survival of cervical cancer patients, but more studies are needed to become a standard.

## Data Availability

The datasets used and/or analysed during the current study available from the corresponding author on reasonable request.

## Abbreviations

OS: Overall Survival
PFS: Progression-free Survival
DFS: Disease-free Survival
C-index: Concordance Index
PNN: Probabilistic Neural Network
ANN: Artificial Neural Network
MLP: Multilayer Perceptron Network
GEP: Gene Expression Programming
SVM: Support Vector Machines
RBFNN: Radial Basis Function Neural Network
RF: Random Forest
LR: Logistic Regression
NB: Naïve Bayes
ML: Machine Learning
DL: Deep Learning
KNN: K-nearest Neighbors
DVH: Dose-volume Histogram
WSI: Whole Slide Image, EL: Ensemble Learning
HL: Hybrid Learning
TCGA: The Cancer Genome Atlas
GEO: Gene Expression Omnibus
SEER: Surveillance, Epidemiology, and End Results
H&E L: Hybrid and Ensemble learning
MAE: Mean Absolute Error
PPV: Positive Predictive Value
NPV: Negative Predictive Value
AUC: Area Under the Curve
HIS: Hospital Information Systems
EHR: Electronic Health Record
PET: Positron Emission Tomography
CT: Computed Tomography
BMI: Body Mass Index
HPV: Human Papillomavirus
